# Establishment of an Experimental Intracerebral Haemorrhage Model for Mass Effect Research using a Thermo-sensitive Hydrogel

**DOI:** 10.1038/s41598-019-50188-y

**Published:** 2019-09-25

**Authors:** Yuhua Gong, Yuping Gong, Zongkun Hou, Tingwang Guo, Jia Deng, Shilei Hao, Bochu Wang

**Affiliations:** 1 Key Laboratory of Biorheological Science and Technology, Ministry of Education, College of Bioengineering, Chongqing University, Chongqing, 400030 China; 2 Department of Ultrasound, Second Affiliated Hospital, Chongqing Medical University, Chongqing, 400010 China; 3 College of Environment and Resources, Chongqing Technology and Business University, Chongqing, 400067 China

**Keywords:** Cerebrovascular disorders, Stroke

## Abstract

The mechanical response of brain tissue closely relates to cerebral blood flow and brain diseases. During intracerebral haemorrhage (ICH), a mass effect occurs during the initial bleeding and results in significant tissue deformation. However, fewer studies have focused on the brain damage mechanisms and treatment approaches associated with mass effects compared to the secondary brain injuries after ICH, which may be a result of the absence of acceptable animal models mimicking a mass effect. Thus, a thermo-sensitive poly (N-isopropylacrylamide) (PNIPAM) hydrogel was synthesized and injected into the rat brain to establish an ICH model for mass effect research. The PNIPAM hydrogel or autologous blood was injected to establish an ICH animal model, and the space-occupying volumes, brain tissue elasticity, brain oedema, neuronal cell death, iron deposition and behavioural recovery were evaluated. The lower critical solution temperature of PNIPAM hydrogel was 32 °C, and the PNIPAM hydrogel had a rough surface with similar topography and pore structure to a blood clot. Furthermore, the ICH model animals who received an injection of PNIPAM and blood produced similar lesion volumes, elasticity changes and mechanically activated ion channel piezo-2 upregulation in brain tissue. Meanwhile, slight iron deposition, neuronal cell death and brain oedema were observed in the PNIPAM hydrogel model compared to the blood model. In addition, the PNIPAM hydrogel showed good biocompatibility and stability *in vivo* via subcutaneous implantation. Our findings show that PNIPAM hydrogel cerebral infusion can form a mass effect similar to haematoma and minimize the interference of blood, and the establishment of a mass effect ICH model is beneficial for understanding the mechanism of primary brain injury and the role of mass effects in secondary brain damage after ICH.

## Introduction

Biomechanics not only plays an important role in the growth and development of brain tissue but also affects the physiology and pathology of the brain. The intracranial mass effect describes when the cranium is occupied by abnormal tissues, which could lead to dysfunction in the patient and even fatal cerebral palsy due to mechanical deformation of the brain. Mechanical deformation of brain tissue has attracted attention in research on many brain diseases (e.g., brain tumour, traumatic brain injury, stroke)^1–3^. Since intracerebral haemorrhage (ICH) is a subtype of stroke, the effect of mass effects on tissue deformation and function should be considered^4^.

ICH has high mortality and morbidity, which includes primary and secondary brain injury according to the progression of the disease^5–8^. Generally, the primary brain injury occurs at the time of haemorrhage, and physical damage of adjacent tissue and formation of the mass effect is a primary injury^1,9^. Mass effect, resulting from the haematoma enlargement and cerebral oedema surrounding haematoma, has been regarded as one of the mechanisms by which ICH may induce ongoing brain injury^4^. On one hand, tissue compression caused by a mass effect can directly induce the neural cell injury^2^. On the other hand, the mechanical effect can also influence the progress of secondary brain injury^1,10^; for example, the hydrostatic pressure promotes red blood cell aggregating and cracking^11^. In past decades, efforts have focused on the research of secondary brain injury factors (thrombin, inflammation and iron)^6,9,12–14^. However, few studies have focused on the mass effect resulting from a haematoma in primary brain injury^2,15^. One of the reasons for stagnation in mass effect research after ICH is the lack of an appropriate experimental animal model.

There are two commonly used rodent ICH models, including infusing autologous whole blood and bacterial collagenase into the striatum^16^. The blood model mimics a single large bleed that occurs in most ICH patients, and the bacterial collagenase injection can disrupt the basal lamina of cerebral blood vessels and cause the blood to leak into the surrounding brain tissue^17^. However, the common ICH animal models cannot effectively avoid the secondary brain insults caused by the physiological response to the haematoma (e.g., inflammation) and the release of clot components (e.g. haemoglobin and iron)^1^. Although a purely mechanical microballoon model has been produced to investigate the mass lesion effects, this microballoon does not truly mimic the irregular shape of a haematoma and shows different mechanical properties compared to blood clots^18^. Therefore, an alternative approach to establish an ICH animal model is necessary to mimic a mass effect haematoma and reduce the interference of blood degradation.

Thermo-sensitive hydrogel, which exists as a free-flowing sol at low temperatures and becomes a polymerized gel at body temperature, has been commonly used in tissue engineering^19,20^. Poly (N-isopropylacrylamide) (PNIPAM), one of the most extensively studied thermo-sensitive polymers, exhibits a lower critical solution temperature of approximately 32 °C in aqueous solution, below which PNIPAM is water-soluble and above which it is water insoluble^21^. Therefore, the PNIPAM hydrogel has the potential to establish an ICH model for mechanical damage research.

The aim of the present study was to establish a rodent ICH model using injectable thermo-sensitive hydrogel to study the mass effect damage caused by ICH. We first detected the thermo-sensitive PNIPAM hydrogel and Young’s modulus values and mechanically activated ion channel Piezo-2 levels of brain parenchyma after the injection of blood or PNIPAM. In addition, we described and compared the lesion volume, neuronal cell death, iron deposition and brain water content between the PNIPAM and blood model. Meanwhile, the biocompatibility of PNIPAM hydrogel was evaluated in the present study. The establishment of a mass effect ICH model helps elucidate the mechanism of primary brain injury after ICH, which could also be used to investigate the role of mass effect in secondary brain damage after ICH.

## Results

### Thermo-sensitive hydrogel synthesis and characterization

**Figure 1:**
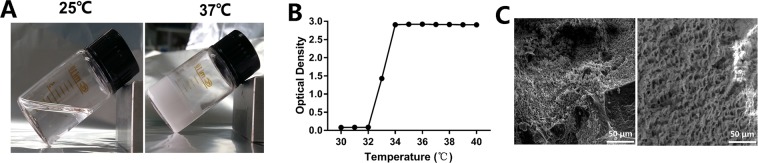
(**A**) Visual inspection of the PNIPAM hydrogel at 25 °C and 37 °C. (**B**) Effects of temperature from 30 to 40 °C on the optical density of PNIPAM hydrogel determined by a microplate reader. (**C**) SEM observation of whole blood clot (left) and PNIPAM hydrogel (right).

A representative visual inspection of the PNIPAM hydrogel at different temperatures was investigated. As shown in Fig. 1A, the appearance of PNIPAM at 25 °C was mobile and transparent, but the solution formed a non-flowing mass and an opaque gel at 37 °C. In addition, the lower critical solution temperature (LCST) was determined by a microplate reader in this study, and the optical density increased sharply from 32 to 34 °C (Fig. 1B).

The morphology of the blood clot and PNIPAM hydrogel were examined using scanning electron microscope (SEM) (Fig. 1C). Pore structure was formed in the blood clot because of the fibrin polymerization, and the rough surface topography was due to blood cell accumulation. In addition, a pore structure and rough surface of the PNIPAM hydrogel was also observed.

### Shear wave elastographic imaging

**Figure 2:**
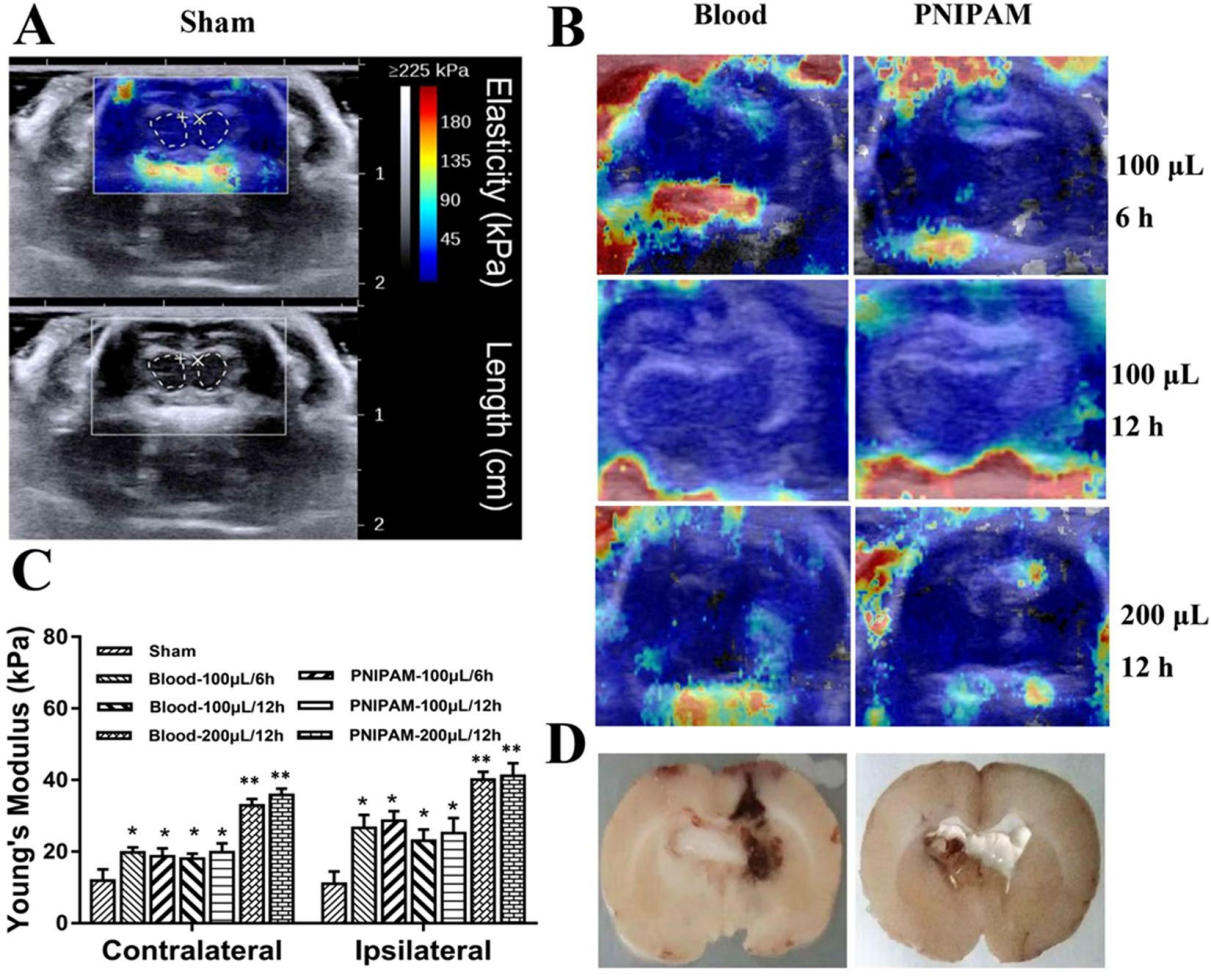
(**A**) Shear wave elastographic image (top) and B-mode image (bottom) of the sham group. The colour legend represents Young’s modulus values (from 0 to 225 kPa). The dashed line position was selected for Young’s modulus values calculation. (**B**) Shear wave elastographic image of PNIPAM and blood models with different times and volumes of cerebral infusion. (**C**) Young’s modulus values of ipsilateral and contralateral hemispheres in each group. Values are means ± SD, *P < *0.05*, **P < *0.01*. (**D**) Tissue slices of ICH models after infusion of 100 µL of blood and PNIPAM hydrogel.

Shear wave elastographic imaging was performed at different times (6 and 12 h) after infusion of different volumes (100 and 200 μL) of PNIPAM and blood. To minimize the interference of the skull and ventricle, the brain parenchyma below the ventricle position was selected as the region of interest for analyses in the combined elastographic model and B-mode images (Fig. 2A, dashed line area), which were chosen for Young’s modulus calculation of average hemisphere and blinded analysis. Figure 2B shows the elastographic imaging of the PINPAM and blood models with different times and volumes of cerebral infusion. The average Young’s modulus values for each hemisphere of sham, PNIPAM hydrogel and blood models were detected (Fig. 2C), and the corresponding slice pictures were depicted to further confirm the space-occupying location (Fig. 2D). A significant difference in average Young’s modulus values was found between the ICH groups (blood and PNIPAM models) and sham group (*P* < 0.05) in both the contralateral and ipsilateral hemispheres, which indicated that the mass effect can be formed by the injection of PNIPAM or blood. Furthermore, the Young’s modulus of ipsilateral hemisphere infused with 200 μL PNIPAM and blood was higher than that infusing with 100 μL (*P* < 0.01), and there was no significant difference in the Young’s modulus of brain tissue between the blood and PNIPAM model, demonstrating that the mass effect caused by PNIPAM injection was similar with that caused by blood infusion. In addition, similar values in the Young’s modulus of brain tissue were observed at 6 and 12 h after injection of 100 μL PNIPAM. The results suggested that a similar mass effect could be formed by injection of the same volume of PNIPAM and blood, and the PNIPAM could supply a stable mass effect after cerebral infusion within 12 h.

### Lesion volume analysis

**Figure 3:**
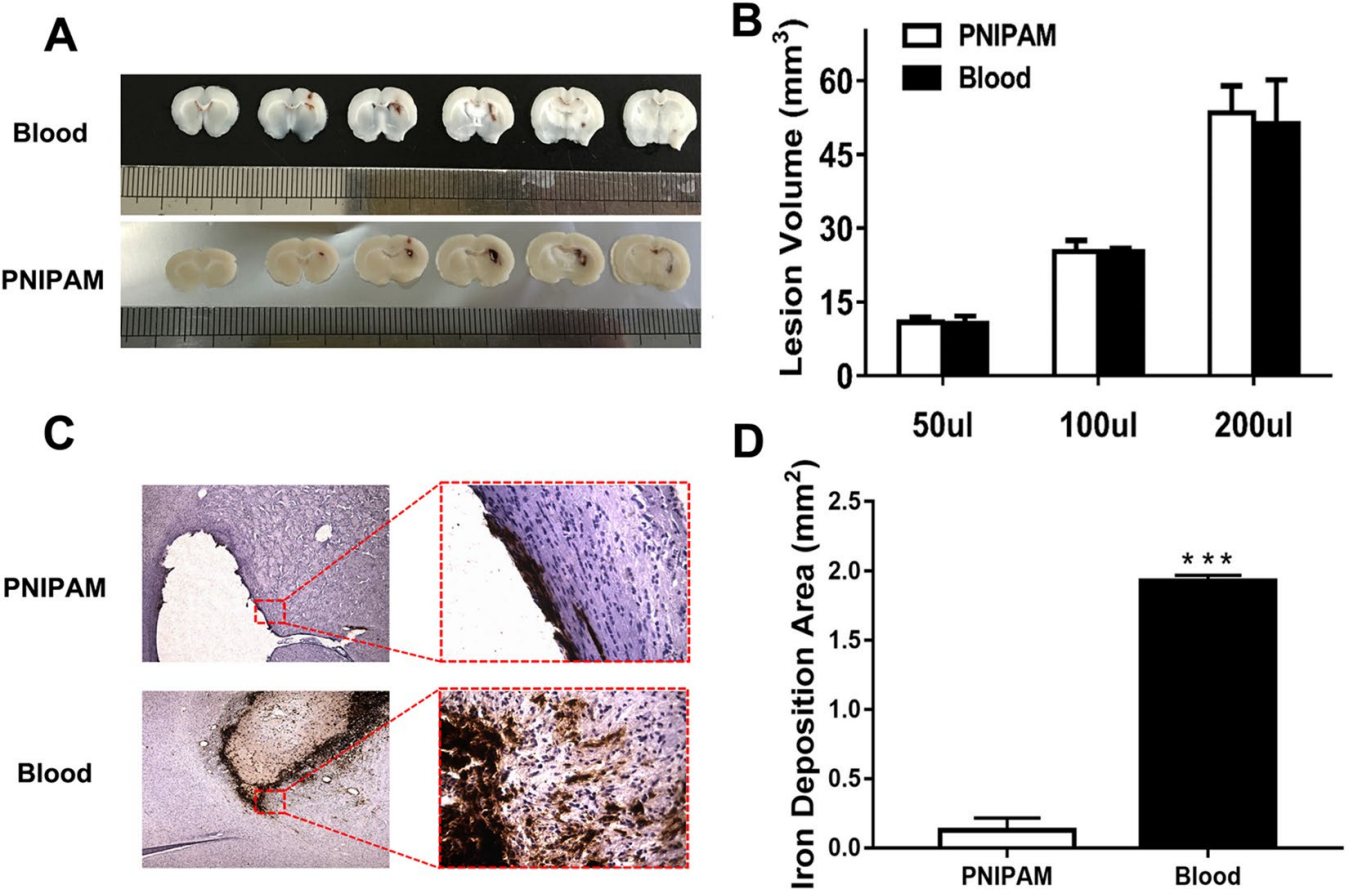
(**A**) Extent of injury in the blood and PNIPAM hydrogel models. (**B**) Lesion volume induced by different injection volumes of PNIPAM or blood injection (50, 100 and 200 µL). Values are means ± SD. (**C**) Iron staining is shown around haematoma. (**D**) Comparison of the results from the iron staining in two models. Values are means ± SD, ***P < 0.001 vs PNIPAM group.

As shown in Fig. 3A, different volumes of tissue lesions were observed by infusion of different volumes of PNIPAM and blood (50, 100 and 200 μL), and an increase in tissue lesion size could be found with the infusion volume of PNIPAM or increased blood (Fig. 3B). However, there was no significant difference in tissue lesion size between the blood and PNIPAM groups, indicating that infusion of PNIPAM hydrogel could form a similar tissue lesion with the blood injection group.

### Iron deposition analysis

The aim to establish a PNIPAM hydrogel model was to discard the secondary injuries caused by blood degradation but form the mass effect similarly as a haematoma. Iron, as the degradation product of blood, could accumulate in the brain parenchyma after blood infusion, which had detrimental effects following ICH. Therefore, the Perl’s staining of tissue slices was performed to evaluate the iron deposition of the PNIPAM hydrogel and blood model. Significantly, more iron deposition was observed in the blood model compared to that in the PINPAM hydrogel model (Fig. 3C). In addition, the iron deposition area was also measured, and the results showed that less iron was deposited in the PNIPAM hydrogel model compared to the blood model (*P* < 0.001) (Fig. 3D).

### TUNEL staining

**Figure 4:**
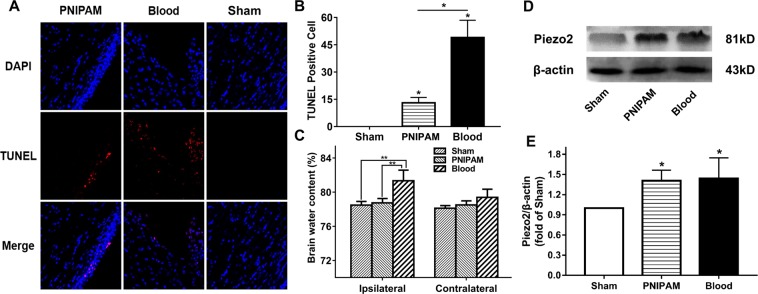
TUNEL staining (**A**) and quantification (**B**) of apoptotic cells at the perihaematoma and cortex at 7 days after ICH. Scale bar = 75 µm. (**C**) Brain oedema formation in rats on day 3 after infusion of blood and PNIPAM hydrogel. (**D**) Western blot assay and quantification (**E**) of Piezo-2 expression in each group. Values are means ± SD, *P < 0.05, **P < 0.01.

TUNEL staining was used to detect the cell death in ICH rats after infusion of blood and PNIPAM hydrogel. As shown in Fig. 4A, TUNEL positive cells were observed around the space-occupying area in both the PNIPAM hydrogel and blood models. In addition, more cell death was found in the blood model compared to the PNIPAM hydrogel model, and quantitative analysis also revealed a significantly increased number of TUNEL positive cells in the blood infusion group than in the PNIPAM hydrogel group (*P* < 0.05) (Fig. 4B).

### Brain oedema analysis

The brain oedema formation in rats was assessed on day 3 after infusion of blood and PNIPAM hydrogel (Fig. 4C). The brain water content of ipsilateral hemispheres (fluid-infused side) in the blood model was significantly higher than that in the sham and PNIPAM model (*P* < 0.01), and the ICH-induced oedema formation was associated with increased iron accumulation in the ipsilateral basal ganglia. In addition, a similar tendency in brain oedema was found in the contralateral hemispheres of ICH rats; notably, no significant difference in the brain water content was found between the blood and PNIPAM hydrogel models.

### Piezo-2 expression analysis

The protein level of mechanically activated ion channel Piezo-2 was investigated at 8 h after ICH (Fig. 4D, E). Piezo-2 level was upregulated dramatically in each ICH group (*P* < 0.05 versus sham), but no significant difference was observed between the PNIPAM and blood injection group (*P* > 0.05).

### Biocompatibility test

**Figure 5:**
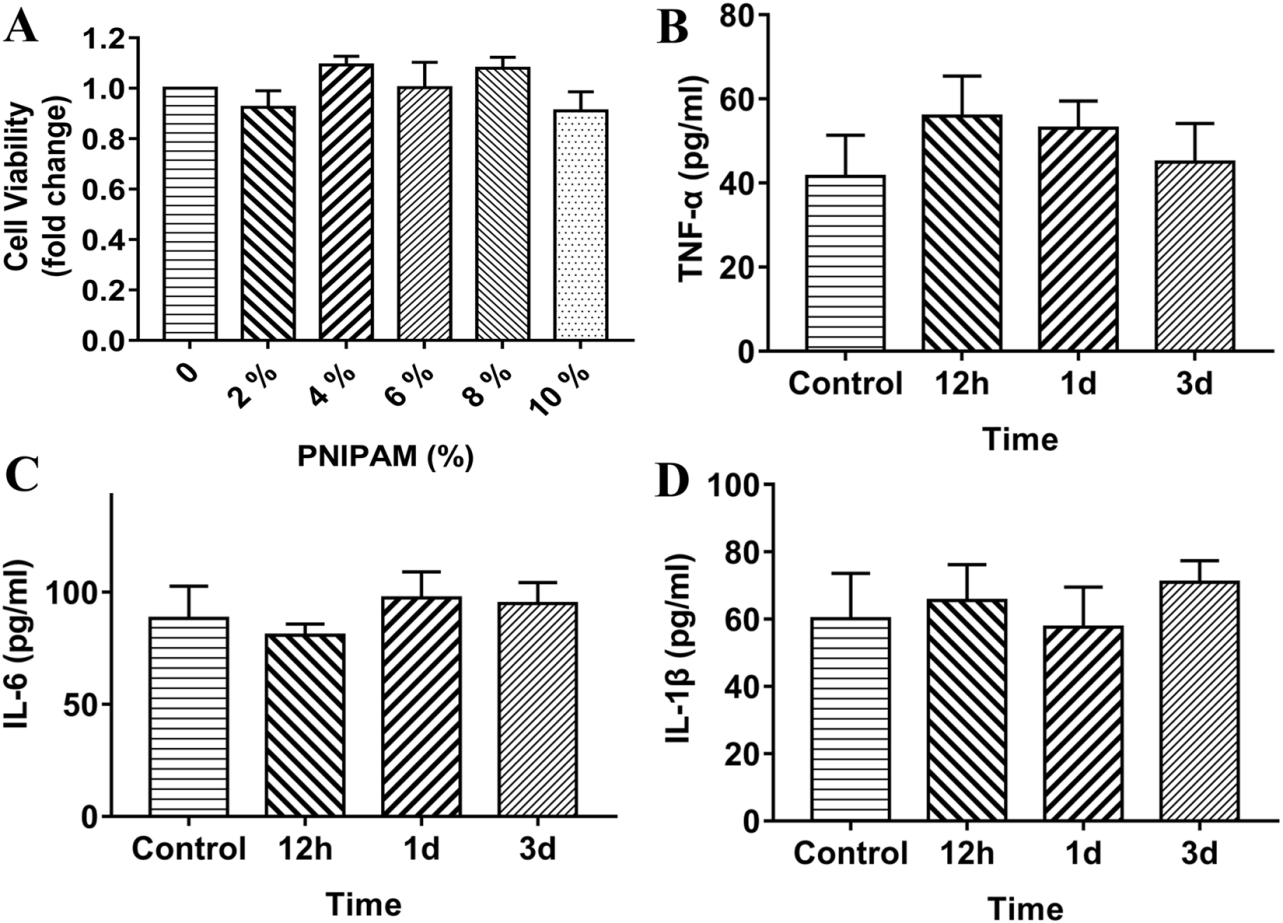
(**A**) Effect of the PNIPAM on neural viability *in vitro*. Effect of PNIPAM on proinflammatory cytokine TNF-α (**B**), IL-6 (**C**) and IL-1β (**D**) expression *in vivo*.

The cytotoxicity of PNIPAM in primary cortical neuron was evaluated with a CCK-8 assay. Compared with the control group, no significant differences in viability were found in the intervention groups (ranging from 2 to 10% PNIPAM) (Fig. 5A). The results indicated that PNIPAM (up to 10%) displayed no obvious cytotoxic effects in primary cortical neurons.

**Figure 6:**
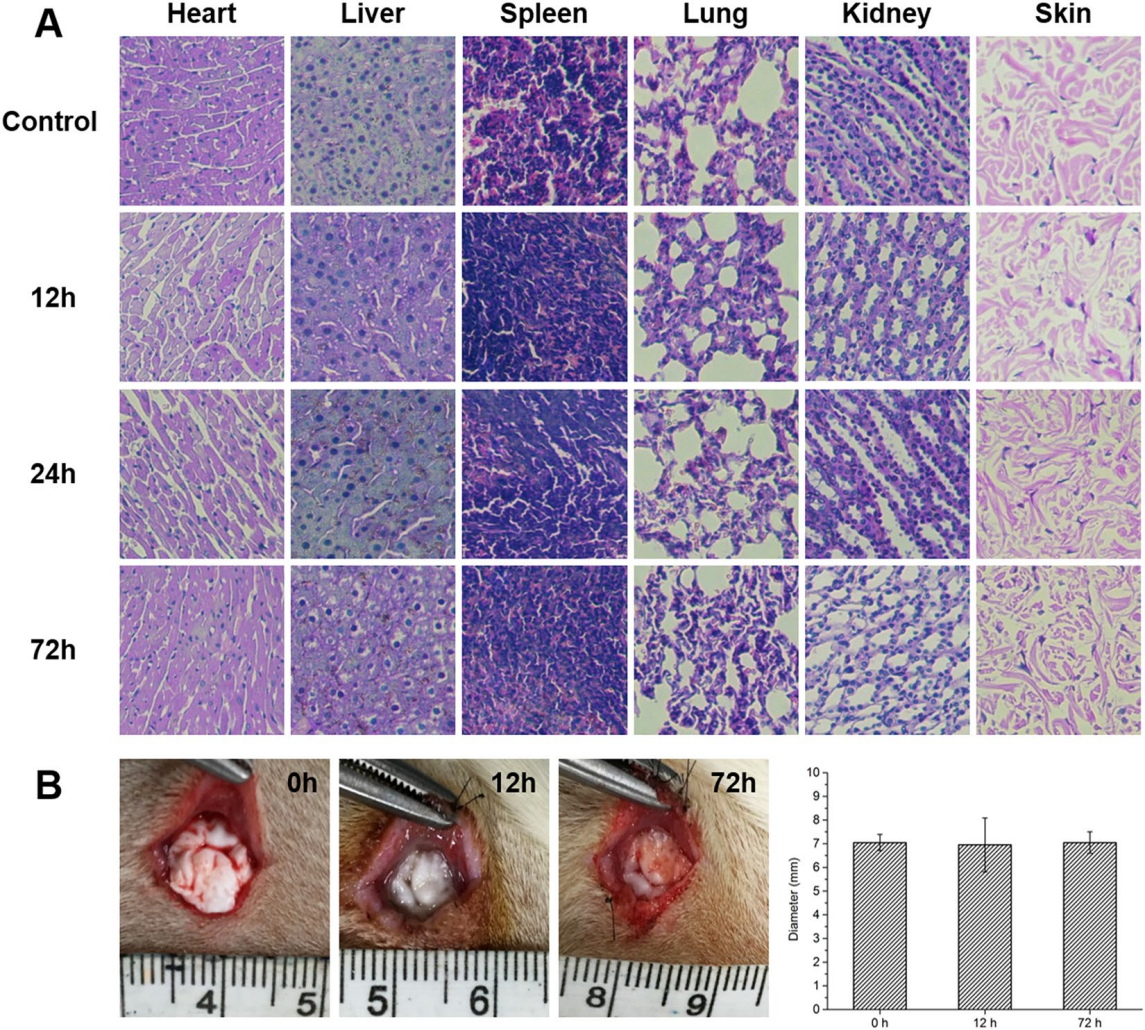
(**A**) H&E staining of main organs (heart, liver, spleen, lung, kidney and skin) after subcutaneous implantation of PNIPAM. (**B**) Images and diameters of PNIPAM after subcutaneous implantation for 0, 12 and 72 h.

ELISA was used to evaluate the effect of PNIPAM on proinflammatory cytokine production before and after subcutaneous implant. The results showed that there were no significant changes in the concentrations of TNF-α, IL-6 and IL-1β between the control and PNIPAM groups (Fig. 5B–D). Moreover, H&E staining of the main tissues (heart, liver, spleen, lungs, kidneys and skins) of normal and implanted rats showed no obvious cell necrosis (nuclear pyknosis, karyorrhexis and karyolysis) after PNIPAM implantation (Fig. 6A), indicating that PNIPAM did not affect the cytokine levels and main tissues morphology after subcutaneous implant. Furthermore, the *in vivo* degradation experiment suggested no significant morphological and dimensional changes in the implanted PNIPAM (Fig. 6B), demonstrating that the material was stable and did not degrade in the body within 72 h.

## Discussion

Brain tissue is in a special mechanical environment because of the spatial limitations of the skull. Many diseases can affect the mechanical properties of brain tissue and cause neurological disorder, especially diseases that can form mass effect, such as cerebral tumor, cerebral cyst and ICH. Although there are few studies on the mass effect of ICH, the volume of hematoma, which is directly related to the mass effect, is recognized as one of the factors affecting clinical prognosis^22^. It has been reported that the mechanical disruption occurred immediately after ICH, and the main reason for the deterioration of pre-period ICH is the mass effect caused by haematoma enlargement^4^. As early as the 1980s, Sinar had established a mechanical microballoon model for the study of mass effect in ICH^23^. Although this model could produce mass effects, significant material and morphological differences with actual haematoma limited its application in preclinical studies.

At present, the models used for ICH research are mainly blood injection and collagenasse injection. Both models can cause the blood clot form in the brain. Numerous studies have shown that the blood clot and its degradation product (hemoglobin, heme and iron) can cause toxic effects on brain tissue including oxidative stress, inflammation and edema^24^. Therefore, the traditional ICH models were unsuitable for mass effect research due to the existing of blood. Thus, it is necessary to establish a novel ICH model, which can mimic the mass effect of haematoma and eliminate blood degradation effects. Although no clinical condition is affected only by mass effect without any secondary damage, and the haematoma may also retract and resolve in long-term metabolism; the establishment of a mass effect ICH model is beneficial to understand the mechanism of primary brain injury after ICH. In addition, this animal model can be used for the development of therapeutic treatments for primary brain injury and to understand the clinical prognosis of early haematoma clearance.

Injectable hydrogels formed by *in situ* chemical polymerization or by the sol–gel phase transition have been applied in tissue engineering, drug delivery, and medical devices. These material systems are flowing and fluid *in vitro*, but rapidly gel after injection under physiological conditions. Among the various injectable hydrogels, thermo-sensitive hydrogels are especially attractive as specific injectable biomaterials due to their spontaneous gelatinization at body temperature and do not require extra chemical treatments^25^. PNIPAM is a widely known thermo-sensitive polymer and an aqueous PNIPAM solution precipitates above 32 °C (LCST)^21^. Therefore, an injectable matrix can be implanted in the brain with minimal surgical wounds, and the modelling process in the present study is similar to the blood and bacterial collagenase models.

Hydrogels are three-dimensional polymeric networks that take in and keep a significant amount of water or biological fluids, and the water content in the PNIPAM hydrogel at room temperature is approximately 75%^26,27^, which is similar with that in the human blood (slightly less than 80%). In addition, the pore structure can be observed due to fibrin polymerization in the blood coagulation process, and the cells accumulated onto the fibres form a rough surface topography. The three-dimensional fibrin network is an important factor in the change of clot viscoelasticity during clot formation. Platelets can only form contractile force in this pore structure, and modulate the fibrin strcutural rigidity by the contractile force^28^. Platelet contraction and fibrin strain stiffening eventually lead to the clot stiffness^29^. A similar pore structure and rough surface can also be found in the PNIPAM hydrogel morphology. Although the surface of PNIPAM hydrogel appears rough, there is no blood cell adhesion. The simple network structure of the PNIPAM hydrogel seems to be advantageous for maintaining a sustained mass effect. Furthermore, PNIPAM is a non-biodegradable polymer^30^, and secondary injuries due to polymer biodegradation can be avoided.

A mass effect in the ICH is a result of the tissue stress response to haematoma. To compare the mass effect in the PNIPAM hydrogel and blood models, brain viscoelasticity was determined to directly indicate the brain tissue stress after the infusion of blood or PNIPAM hydrogel. Ultrasound-based transient elastography by shear wave imaging enables the real-time visualization of transient shear waves propagating in the brain^31^, and the local speed of shear wave propagation is proportional to the Young’s modulus^32^. The increased Young’s modulus values have also been found in mild traumatic brain injury of rodent, which are result from the edema and hemorrhage^33^. Hemorrhage and edema are more prominent in the ICH process, suggesting that Young’s modulus values variation may be involved in the ICH. In this study, we compared the Young’s modulus of the brain parenchyma in the PNIPAM hydrogel and blood models. An increase in Young’s modulus values of brain tissue was noted after the infusion of PNIPAM hydrogel and blood, but there was no significant difference in Young’s modulus of rat brain between the two models, suggesting that the PNIPAM hydrogel model had a similar mass effect with the blood model.

The degradation of hematoma after ICH mainly includes microglia/macrophages phagocytosis and erythrocyte lysis. Both ways can lead to the accumulation of free iron through different metabolic pathways^24^. Iron toxic is mainly through the Fenton reaction to produce reactive oxygen species, which can lead to structural change of membranes, lipids, proteins and nucleic acids^34^. Iron-induced neurotoxicity effect has been studied in a variety of diseases, including Parkinson’s disease, Friedreich’s ataxia, traumatic brain injury and hemorrhagic stroke^24,35^. Brain iron overload plays a detrimental role in brain injury after ICH, which causes brain oedema in the acute phase and brain atrophy later^36^. Preclinical studies of ICH have clearly demonstrated the effectiveness of iron overload intervention^37^. To examine the mass effect after ICH, it is necessary to avoide the toxic effect of iron. Therefore, this study measured the Perls’ deposition of this new mass effect research model. The ideal mechanical ICH animal model can mimic the mass effect of haematoma but minimize the influence of blood breakdown products because bleeding cannot be avoided during cerebral infusion operation. In the present study, a significant decrease in iron deposition had been found in the PNIPAM hydrogel model compared to the blood model. Meanwhile, marked brain oedema and neuronal cell death were observed in the blood model but not in the PNIPAM hydrogel model.

## Conclusions

Although there are no clinical conditions that are affected only by mass effect without any secondary damage, the establishment of a mass effect ICH model is beneficial to understand the mechanism of primary brain injury and the role of mass effect in secondary brain damage after ICH. Thus, a novel PNIPAM hydrogel infusion ICH animal model was established for ICH mass effect research. PNIPAM hydrogel supports a similar structure and water content with blood, and PNIPAM hydrogel injection caused a similar mass effect compared to haematomas. In addition, less iron deposition was observed in the PNIPAM hydrogel model, which resulted in slight brain oedema and neuronal cell death.

## Materials and Methods

### Thermo-sensitive hydrogel synthesis and characterization

The PNIPAM hydrogel was prepared following a previous study^38^. Briefly, 1 g of N-isopropylacrylamide (NIPAM) monomer (Damas-beta, Shanghai, China) was dissolved in 19 mL of distilled water, and 1 mL of ammonium persulfate solution with a concentration of 1.2 × 10^−3^ g/mL was added to the NIPAM solution to induce polymerization. The mixture was magnetically stirred in a water bath at 60 °C for 30 minutes in a nitrogen environment, and the reaction product was a milky white liquid, which transforms to a clear gelatinous liquid at room temperature.

The thermo-sensitive analysis of the PNIPAM hydrogel was performed using a microplate reader (ELx808, Biotech, USA) ranging from 30 °C to 40 °C, and the absorbance of each well was read at the test wavelength of 570 nm. An average of the three values was taken as the LCST of the PNIPAM hydrogel.

The morphology of the PNIPAM hydrogel and rat blood clot was observed in the present study. The PNIPAM hydrogel and rat blood clot were frozen at −80 °C and then lyophilized by freeze-drying. The dried samples were sputter-coated with gold and examined using SEM (Nova NanoSEM 400, FEI).

### ICH animal model establishment

Animal procedure protocols were endorsed by the Institutional Animal Care and Use Committee of the Third Military Medical University, China. All experiments were performed according to the guidelines and regulations of Third Military Medical University, China. This study was performed using male Sprague-Dawley (SD) rats weighing 250–300 g, which were obtained from the Experimental Animal Center of the Third Military Medical University. Rats were housed in a constant temperature room (22–25 °C, light/dark cycle) with free access to food and water during the research. A total of 156 rats were included in this study. Data were reported on 144 animals. Twelve rats were excluded from this study due to a lack of haematoma formation (5) or death (7). Rats were randomly divided into groups (sham, PNIPAM hydrogel, or blood) for the experimental animal studies.

Rats were anaesthetized with chloral hydrate (300 mg/kg), and body temperature was maintained at 37 °C using a heating pad. Rats were fixed in a stereotaxic frame, and a cranial burr hole was drilled at the following relative stereotactic coordinates: 5.5 mm ventral to the surface, 0.2 mm anterior and 2.5 mm lateral to bregma. Autologous blood or prepared PNIPAM hydrogel was injected into the right basal ganglia by a 26-gauge needle at a uniform speed (10 μL/min)^39^. Autologous blood was collected from the orbit with a microscopic blood collection tube and the prepared PNIPAM hydrogel was sterilized with ultraviolet radiation in the ultra-clean platform. The injection volume of blood or PNIPAM is 100 μL unless otherwise specified. Sham-operated group only needles to the same depth at the same position. After injection, the needle was reserved in position for more 10 minutes and was then gently withdrawn. The skull hole was closed with bone wax, and the surgical skin was sutured. The sham group had the same cranial burr hole and only intracerebral needle insertion. Each part of the experiment selected different time points for analysis according to the pre-experiment and related preclinical research literature.

### Shear wave elastographic imaging

Shear wave elastographic imaging can reflect the Young’s modulus of tissue based on the propagation velocity of the shear wave in the tissue. Young’s modulus value is positive correlated with tissue hardness. Shear wave elastographic imaging is a powerful tool for the assessment of elasticity changes in brain tissues due to the mass effect of haematoma and PNIPAM hydrogel^31,40^. The rats received bilateral craniectomies after infusion of autologous blood or PNIPAM hydrogel (n = 3 per group). The cranial window was enlarged to approximately 10 mm × 10 mm (typically taking the bregma as coordinate, from +3 mm to −7 mm and 5 mm to each lateral). The cerebral shear wave elastographic image of the ICH rats was performed on a linear SL15-4-MHz transducer and an FDA-approved multiwave ultrasound system (Aixplorer, SuperSonic Imagine, France). The transducer’s imaging frequency was 12 Hz. First, the conventional B-mode scans of the brain were acquired to determine the location of the haematoma. Then, shear wave elastographic images were collected immediately with an elasticity range of 0 to 225 kPa to minimize the gross imaging artefacts of the skull and surrounding tissue.

Ten images of each rat were collected. Three images with an identifiable B-mode image (including identified haematoma) and minimal elasticity image artefacts were selected randomly for the correlation analysis. Subsequently, rats were euthanized, and the brains were harvested for anatomical analysis. The brains were serial sliced into 1 mm thickness and photographed with a digital camera.

### Lesion volume analysis

Different volumes of autologous blood or PNIPAM hydrogel (50, 100 and 200 μL) were injected to establish the different lesion degrees of the ICH models (n = 3 per group). ICH rats were allowed to live 1 day and then were perfused with 0.9% saline through the left ventricle followed by 4% paraformaldehyde. Brains were harvested and immersed in 4% paraformaldehyde for 24 h and then serial sliced into 6 or 8 coronal sections of 1 mm thickness at the needle site of the coordinate centre^41^. The photos of the slices were processed with ImageJ software. The lesion volume was expressed as the sum of the lesion area of each section and multiplied by the slice thickness.

### Histology

Animals were anaesthetized at days 3 after injection of blood or PNIPAM hydrogel (n = 3 per group). Rats were perfused through the left ventricle with 0.9% saline and 4% paraformaldehyde. Brains were removed and immersed in 4% paraformaldehyde for 24 h. Samples were embedded in an optimal cutting temperature compound, and serial coronal sections were cut with a microtome.

Cell death of coronal frozen slices was detected using an *in situ* cell death detection kit (Roche Diagnostic), and the procedure was performed according to the manufacturer’s instructions. After ICH, TUNEL-positive cells can be detected from 6 hours to more than 2 weeks^42^. The number of positive and total cells was counted at 200× magnifications in the perihaematoma region and the ipsilateral cortex (n = 3 per group).

Iron Perl’s staining was performed to evaluate iron deposition of microglia/macrophages in the perihaematoma and surrounding tissue^43^. The deposition of non-heme iron in brain tissue caused by the lysis of erythrocytes reached a plateau at day 3 after ICH^44^. Three sections of each rat with maximum haematoma diameter were selected (n = 3 per group), and brain sections were incubated in Perl’s solutions (1:1, 5% potassium ferrocyanide and 5% HCl) for 45 min, washed using distilled water, and then incubated in 0.5% diamine benzidine tetrahydrochloride with nickel for 60 min. The iron deposition was quantified by measuring the staining area using ImageJ.

### Brain water content

Brain oedema was determined by the wet–dry weight ratio method as described previously^43^. After PNIPAM hydrogel and blood infusion for 3 days, rats were euthanized (n = 3 per group). The brain tissue was divided into 2 hemispheres along the midline. The brain tissue was wrapped in foil and weighed by an electronic analytic balance (ME204E, Mettler Toledo, China) to obtain the wet weight. Samples were then dried in a gravity oven at 100 °C for 24 h to obtain the dry weight. Brain water content (%) was calculated as ([wet weight − dry weight]/ wet weight) × 100%.

### Western blotting analysis

Animals were anaesthetized at 8 hours after ICH. An ipsilateral basal ganglia sample was harvested to determine Piezo-2 protein levels (n = 3 per group). Western blotting was performed as described previously^2^. Each sample that contained 50 μg protein was separated by SDS-polyacrylamide gel electrophoresis. Proteins were blotted onto a PVDF membrane and incubated with polyclonal rabbit anti-FAM38B (Abcam; 1:500 dilution) and polyclonal rabbit anti-β-actin (Proteintech; 1:1000 dilution). The secondary antibody was goat anti-rabbit IgG (Beyotime; 1:1000 dilution). The antigen-antibody complexes were visualized by the ECL electrochemiluminescence and quantified by Quantity-one. Representative strips were proceeded through “Microsoft Office PowerPoint 2016”.

### Biocompatibility test

#### Cell viability assay

Primary cortical neuronal culture was performed according to a previous method^2^. The cytotoxicity of PNIPAM in neurons was determined by a cell proliferation assay (CCK-8, Dojindo Laboratories, Kumamoto, Japan). The cells (5 × 10^3^ cells/well) were seeded on a 96-well cell culture plate in an incubator for 24 h and treated with different concentrations of PNIPAM (2, 4, 6, 8, 10%, v/v) for another 24 h. Cells were then treated with CCK-8, and the absorbance at 490 nm was determined using a microplate reader.

#### *In vivo* biocompatibility test

Subcutaneous implantation of materials was usually used to detect the *in vivo* biocompatibility^45^. Rats were anaesthetized, and a 1.2 cm incision was made on the dorsal skin. After incubating at 37 °C, PNIPAM was made into one disk shape of approximately 0.8 mm in diameter and subcutaneously inserted into the incision. The subcutaneous implant pictures were continuously photographed at 0, 12 and 72 h to observe the degradation of PNIPAM (n = 3 per group).

The sera of these rats were collected by centrifugation and used for proinflammatory cytokine detection. TNF-α, IL-6 and IL-1β levels were tested using enzyme-linked immunosorbent assay kits according to the manufacturer’s instructions (Neobioscience Technology Co., Ltd, China). Meanwhile, haematoxylin and eosin (H&E) staining was performed according to standard protocols and used for evaluating the toxicity of PNIPAM to the main organs (heart, liver, spleen, lung, kidney and skin). The main organs were harvested at 12, 24 and 72 h post-subcutaneous implantation.

### Statistical analysis

All data were presented as the means ± SD. A paired-samples T-test was used for two groups, and a two-way ANOVA was used for more than two groups with SPSS 19.0 software. Differences with a value of *P < 0.05* were considered statistically significant.
